# Supplementary material for: Combined Liquid Biopsy Methylation Analysis of CADM1 and MAL in Cervical Cancer Patients
Source: Cancers (Basel). 2022 Aug 16;14(16):3954. doi: 10.3390/cancers14163954 (PMC9406083; doi:10.3390/cancers14163954)
Supplement: Supplementary file 1 [file cancers-14-03954-s001.zip › cancers-1831997-supplementary.pdf]

Combined Liquid Biopsy Methylation Analysis of CADM1 and MAL in Cervical Cancer Patients

Supplements:

Table S1: Clinical characteristics of cervical dysplasia patients.

		Count (N=52)	%
Age [a]	≤35	20	38.5%
	>35	32	61.5%
Grade of dysplasia	CIN 2	10	19.2%
	CIN 3	42	80.8%
Cytological status (Pap classification)	I	1	1.9%
	II-a	6	11.5%
	II-p	1	1.9%
	II-g	1	1.9%
	III-p	2	3.9%
	IIID1	4	7.7%
	IIID2	9	17.3%
	IVa-p	24	46.2%
	IVa-g	1	1.9%
	IVb-p	1	1.9%
	V-p	2	3.9%
HPV status	Positive for one subtype	21	40.4%
	Positive for multiple subtypes	28	53.8%
	Negative	3	5.8%
Most frequent subtypes	HPV 16	24	46.2%
	HPV 18	5	9.6%
	HPV 31	4	7.7%
HPV vaccination	Not vaccinated	42	80.8%
	Vaccinated [b]	6	11.5%
	Unknown	4	7.7%

a. Mean age of onset in Germany is 35.[1]
b. All 6 patients are positive for HPV:
4 patients are positive for an HPV subtype against which they have not been vaccinated; 1 patient was vaccinated later than the recommended timing; 1 patient has multiple HPV infections due to a history of AML and corresponding immunosuppressive phase.

Table S2: Clinical characteristics of cervical cancer patients.

		Count (N=24)	%
Age [a]	≤55	18	75.0%
	>55	6	25.0%
TNM	T1aN0M0	4	16.7%
	T1bN0M0	5	20.8%
	T2bN0M0	2	8.3%
	T1-4N1M0	9	37.5%
	T1-4N0-1M1	4	16.7%
FIGO stage	IA	4	16.7%
	IB	5	20.8%
	IIB	2	8.3%
	IIIC	9	37.5%
	IVB	4	16.7%
Tumor grade	G1	2	8.3%
	G2	14	58.3%
	G3	6	25.0%
	Unknown	2	8.3%
Histology	Squamos cell carcinoma	22	91.7%
	Adenocarcinoma	2	8.3%
HPV status	Positive	17	70,8%
	Negative	1	4,2%
	Unknown	6	25.0%
Point of blood sampling	Before therapy [b]	12	50.0%
	Under ongoing therapy [c]	12	50.0%

a. Mean age of onset in Germany is 55. [1]
b. 7 initial diagnoses, 2 initial diagnoses after conization (local R1), 3 relapse diagnoses.
c. 5 under radiochemotherapy, 5 under chemotherapy, 2 between tumor surgery (local R0) and radiochemotherapy.

Table S3: CADM1, MAL and CADM1 and/or MAL methylation status of blood samples from 24 cervical cancer patients in relation to histological subtype.

	Histology			
	Squamous cell carcinoma		Adenocarcinoma	
	Count (N=22)	%	Count (N=2)	%
CADM1	17	77.3%	1	50.0%
MAL	10	45.5%	0	0.0%
CADM1 and/or MAL	19	86.4%	1	50.0%

Table S4: CADM1, MAL and CADM1 and/or MAL methylation status of blood samples from 24 cervical cancer patients in relation to tumor grade.

	Tumor grade							
	G1		G2		G3		Unknown	
	Count (N=2)	%	Count (N=14)	%	Count (N=6)	%	Count (N=2)	%
CADM1	0	0.0%	11	78.6%	5	83.3%	2	100.0%
MAL	1	50.0%	3	21.4%	5	83.3%	1	50.0%
CADM1 and/or MAL	1	50.0%	11	78.6%	6	100.0%	2	100.0%

Table S5: CADM1, MAL and CADM1 and/or MAL methylation status of blood samples from 24 cervical cancer patients in relation to HPV status.

	HPV status					
	Positive		Negative		Unknown	
	Count (N=17)	%	Count (N=1)	%	Count (N=6)	%
CADM1	14	82.4%	1	100.0%	3	50.0%
MAL	8	47.1%	0	0.0%	2	33.3%
CADM1 and/or MAL	16	94.1%	1	100.0%	3	50.0%

References

1. Barnes, B.; Kraywinkel, K.; Nowossadeck, E.; Schönfeld, I.; Starker, A.; Wienecke, A.; Wolf, U. *Bericht zum Krebsgeschehen in Deutschland 2016*; Robert Koch-Institut: Berlin, Germany, 2016..
